# CRISPR typing and phage content of colonizing Group B *Streptococci* from healthy Egyptian women

**DOI:** 10.3389/fcimb.2025.1636071

**Published:** 2025-08-28

**Authors:** Sarah Shabayek, Dorina Haider, Verena Vogel, Uzma Basit Khan, Dorota Jamrozy, Elita Jauneikaite, Kirsty LeDoare, Stephen Bentley, Barbara Spellerberg

**Affiliations:** ^1^ Department of Microbiology and Immunology, Faculty of Pharmacy, Suez Canal University, Ismailia, Egypt; ^2^ Institute of Medical Microbiology and Hygiene, University Hospital Ulm, Ulm, Germany; ^3^ Parasites and Microbes, Wellcome Sanger Institute, Hinxton, United Kingdom; ^4^ Oxford Centre for Microbiome Studies, Kennedy Institute of Rheumatology, University of Oxford, Oxford, United Kingdom; ^5^ Department of Metabolism, Digestion and Reproduction, Institute of Reproduction and Developmental Biology, Imperial College London, London, United Kingdom; ^6^ Centre for Neonatal and Paediatric Infection, Maternal and Neonatal Vaccinology Group, City St. Georges University of London, London, United Kingdom

**Keywords:** GBS (Group B Streptococcus), CRISPR, Africa, epidemiology, phage (bacteriophage)

## Abstract

**Background:**

*Streptococcus agalactiae* or Group B *Streptococcus* (GBS) causes serious infections in neonates with a particularly high burden of disease in Africa. Maternal vaginal colonization is the primary source of neonatal transmission. Molecular surveillance of the maternal GBS population is crucial for informing maternal vaccine development and monitoring of the global circulation of GBS clones.

**Methods:**

The current study analyzes the structure and diversity of the clustered regularly interspaced palindromic repeat (CRISPR)-associated (Cas) system and phage content in colonizing GBS isolates collected from healthy pregnant women from Ismailia, Egypt. The isolates were characterized by whole-genome sequencing within the global JUNO project.

**Results:**

CRISPR arrays and phages were detected in a vast majority of GBS isolates. A strong congruence was observed between multilocus sequence typing (MLST), CRISPR profile, and phage content. Region-specific sequence types (STs) observed only in Africa were distinguishable from other lineages.

**Conclusions:**

CRISPR typing is a promising low-cost tool for investigating the population structure of GBS clones, particularly in middle- and low-income countries.

## Introduction

The clustered regularly interspaced palindromic repeat (CRISPR)-associated (Cas) system is an adaptive immune system of prokaryotes providing protection against invading nucleic acids associated with mobile genetic elements (MGEs), such as phages and plasmids (Barrangou et al., 2007; Lopez-Sanchez et al., 2012). The CRISPR-Cas locus typically consists of highly conserved direct repeats (DR) separated by non-repetitive invader-targeting spacer sequences of a similar length and genes encoding CRISPR-associated proteins (Cas proteins) (Kunin et al., 2007; Lopez-Sanchez et al., 2012). Spacer cleavage derived from invading DNA is mediated by the direct hybridization of small CRISPR RNAs (crRNAs), which act in conjunction with Cas proteins (Deltcheva et al., 2011). Many of the repeat-spacer arrays are flanked by a leader sequence on one side and a trailer sequence on the other. CRISPR arrays represent a chronological archive of past invaders as recently acquired spacers are integrated at the leader proximal and sorted in a linear, time-oriented manner. Polarized acquisition of new spacers is concomitant to the duplication of a DR (Barrangou and Dudley, 2016; Beauruelle et al., 2017).

*Streptococcus agalactiae* or group B streptococcus (GBS) is a pathobiont that colonizes the gastrointestinal and genitourinary tracts of healthy humans (Verani et al., 2010; Brokaw et al., 2021). GBS is a leading cause of sepsis, meningitis, and pneumonia in neonates (Verani et al., 2010; Brokaw et al., 2021). Maternal carriage is the principal route of GBS vertical transmission to newborns (Verani et al., 2010; Brokaw et al., 2021). It can also cause severe infections in the elderly and non-pregnant adults (Verani et al., 2010; Brokaw et al., 2021). Two CRISPR-Cas systems have been described in GBS, a type II-A system, named CRISPR1 and a type I-C system named CRISPR2. While the CRISPR1 locus is ubiquitous and functional, the CRISPR2 locus is present in a few strains and is less active (Lopez-Sanchez et al., 2012). The CRISPR1 locus is extremely diverse and highly dynamic with continuous spacer acquisitions and duplications as well as spacer deletions. It is typically composed of highly conserved DRs of 36 bp, which are interspaced with spacers of 30 bp. Conversely, the CRISPR2 locus is less diverse and less dynamic with DRs of 32 bp (Lopez-Sanchez et al., 2012).

Prophages, integrated into bacterial chromosomes, play crucial roles in virulence, pathogenicity, and the regulation of bacterial ecology and colonization (Penadés et al., 2015; Davies et al., 2016). CRISPR-Cas systems control phage acquisition (Barrangou et al., 2007), whereas prophages are associated with horizontal gene transfer in bacteria, enhancing genomic diversity and accounting for up to 10% of all strain-specific genes in GBS (Tettelin et al., 2005; Juhas, 2015). Lysogeny is common among a variety of bacterial species; in GBS, it was first described in 1969 in strains of bovine origin (Khan et al., 2024). While the majority of GBS isolates contain one or more prophages, differences in prophage distribution among GBS serotypes and clonal complexes have been reported reflecting GBS population heterogeneity (Domelier et al., 2009; Salloum et al., 2011; van der Mee-Marquet et al., 2018).

The population structure of GBS has been investigated using different typing methods including serotyping and multilocus sequence typing (MLST) (Jones et al., 2003). Although serotyping is relatively less expensive and easy to perform in the laboratory, its discriminating power is insufficient for a detailed comparison of isolates (Sabat et al., 2013; Beauruelle et al., 2021) and has in many laboratories been replaced by molecular genetic typing methods. MLST is currently the standard for GBS typing (Beauruelle et al., 2021). However, a major disadvantage of MLST is that it is expensive, time-consuming, and labor-intensive (Radtke et al., 2010; Sabat et al., 2013; Beauruelle et al., 2021). The vast majority of GBS isolates harbor a CRISPR type II-A system (Lopez-Sanchez et al., 2012). A typing method based on CRISPR array analysis with high discriminatory power has been shown to be efficient in separating GBS isolates (Lopez-Sanchez et al., 2012). This genotyping approach discriminates isolates according to the CRISPR1 spacer content of the type II-A system based on similarities between spacers. Unlike MLST, CRISPR typing is an easy and low-cost method (Beauruelle et al., 2021). Recent studies suggested CRISPR typing as a promising alternative to MLST (Beauruelle et al., 2021) especially in low-resource settings.

While there is a scarcity of GBS epidemiological data for many African countries, this region is particularly indicated with a high burden of GBS vaginal carriage and disease (Shabayek et al., 2022). One of the main reasons for the scarcity of data is the high cost of GBS typing methods (Capan et al., 2012; Russell et al., 2017). The low-cost CRISPR approach has been proposed as a useful tool with high discriminatory power for GBS typing in middle- and low-income countries (Beauruelle et al., 2021) and may be especially useful in view of the introduction of GBS vaccines in the near future.

Our work builds on a previous study (Shabayek et al., 2022) where the MLST types of 90 colonizing Egyptian GBS isolates from pregnant and non-pregnant women have been described. The current study focuses on the analysis of the structure and diversity of CRISPR-Cas profiles and their use for epidemiological typing in these 90 Egyptian GBS isolates using whole genome sequencing (WGS) within the JUNO project (Project JUNO, 2021). Furthermore, the diversity of GBS prophages found in the Egyptian GBS isolates, including their distribution across different STs and correlation with CRISPR1 array clusters, was analyzed.

## Materials and methods

Bacterial genomic DNA was extracted using the GenElute Bacterial Genomic DNA Kit (Sigma-Aldrich, St. Louis, MO, USA) according to the manufacturer’s instructions. Whole-genome sequencing (WGS) was performed at the Wellcome Sanger Institute as part of the global GBS surveillance study JUNO [https://www.gbsgen.net/ (accessed on 2 August 2024)] (Project JUNO, 2021). Briefly, WGS libraries were prepared using the NEB Ultra II library kit on an Agilent Bravo liquid handling platform, and the sequencing was performed on the Illumina HiSeq platform with 150-base-pair read length. Short-read data are available from the European Nucleotide Archive (see **Supplementary Table S1** for sample accession numbers). Genomes were assembled using the SPAdes genome assembler (version 3.14.0) (Bankevich et al., 2012). Subsequently, multilocus sequence typing (MLST) was performed with the assembled genomes by utilizing the PubMLST database (Jolley et al., 2018; Shabayek et al., 2022). WGS data were analyzed for CRISPR loci using CRISPRCasFinder (Couvin et al., 2018; Makarova et al., 2020). Only CRISPR arrays with evidence level 3 or 4 were retained for further analysis. The rest were discarded as CRISPR arrays having evidence levels 3 and 4 are considered highly likely candidates, whereas evidence levels 1 and 2 indicate potentially invalid CRISPR arrays (Couvin et al., 2018; Makarova et al., 2020). The evidence levels 1-4 are a rating system to discriminate fake CRISPR-like elements from true CRISPRs. The lowest evidence level (rated 1) is given to short candidate arrays made of one to three spacers as these often do not correspond to CRISPRs. Putative CRISPR arrays with at least four spacers are assigned to levels 2–4 based on an EBcons (entropy-based conservation) index, which is produced through an algorithm to measure CRISPR repeat conservation and degree of similarity between spacers. To assess the conservation of CRISPR-Cas type II-A systems in all African countries, the presence of the *csn2* gene, a signature gene of CRISPR-Cas type II-A systems (Makarova et al., 2020), was analyzed in publicly available genomes of GBS strains isolated in nine other African countries (study references: PRJEB11000, PRJEB18603, PRJEB8986, PRJEB41294, PRJEB43245, PRJEB44246, PRJEB46377, PRJNA63204, PRJNA556442, PRJNA986888, PRJNA315969, PRJNA407943, and PRJNA479604; for ENA accession numbers, see **Supplementary Table S2**). *In silico* PCR was conducted using the following primers: 5'ATGATCAAGATTAATTTTCC3' and 5'TTATACCATATTTTCGCCTA 3'. Graphic presentation, visualization, and alignment of CRISPR loci were performed using CRISPRViZ (Nethery and Barrangou, 2019). A phylogenetic tree of CRISPR1 loci was built using the Interactive Tree Of Life (iTOL) (Letunic and Bork, 2024) online tool (https://itol.embl.de). A Newick file of the initial tree was generated by the Molecular Evolutionary Genetics Analysis (MEGA) software version 11 (Tamura et al., 2021) through multiple-sequence alignment of CRISPR1 sequences using ClustalW and the neighbor-joining algorithm. This was then uploaded to the online iTOL to build a circular phylogenetic tree. The assembled genomes were analyzed for prophage sequences using PHASTEST (Wishart et al., 2023). Only predicted prophage regions with the completeness level “intact” were considered for further analysis. Prophage regions with completeness level “incomplete” and “questionable” were discarded. The Basic Local Alignment Search Tool (BLAST) (Altschul et al., 1990) was used to identify homologous regions. Prophage sequences were aligned using Qiagen CLC Main Workbench 7.7.3 with default parameters (gap open cost: 10; gap extension cost: 1,0). Qiagen CLC Main Workbench 7.7.3. was used for bootstrap analysis (100 replicates) and construction of the phylogenetic tree (tree construction method: neighbor joining algorithm; nucleotide distance measure: Jukes-Cantor). Prophage sequences were assigned into clusters A-P according to the phylogenetic tree, and clusters were aligned separately using the Clustal Omega tool (Madeira et al., 2024). Clustering was retained, when the percent identity of the aligned sequences within each cluster was at least 75% (**Table 1**; **Supplementary Table S3**).

**Table 1:** Prophage clusters and similarity to previously characterized phages.

Prophage group	No. of prophages in genomes	Identity within Prophage group	Phage hit	Coverage	Identity
A	14	100%	Javan55	66%-67%	100%
B	9	100%	Javan51	66%-80%	100%
C	3	78%-89%	phiStag1_20280_6_181	69%-95%	92%-100%
D	4	100%	Javan29	68%	100%
E	11	100%	Javan55	67%	100%
F	4	100%	Javan29	68%	100%
G	2	100%	No match	-	-
H	4	87%-100%	Not consistent	-	-
J	3	99%-100%	Javan39	70%-85%	100%
K	3	96%-100%	vB_Sags-UPM1	52%-53%	90%-91%
L	4	98%-100%	Javan23	68%-94%	99%
M	3	97%-100%	phiGBSVK-D_GBSInt2.2	82%-87%	99%-100%
N	2	96%	LF2	43%-78%	90%
O	3	98%-100%	phiGBSVK-F2_GBSInt11.1	70%-90%	99%
P	2	100%	No match	-	-

## Results

We investigated the CRISPR1 and CRISPR2 loci distribution in a collection of 90 whole-genome sequenced colonizing GBS isolates from healthy Egyptian women. Using CRISPRCasFinder, the CRISPR1 sequence was detected in 88 isolates, confirming its ubiquitous nature and conserved structure in GBS. The complete sequence was generated for each isolate, enabling the analysis of spacers, direct repeats, leaders, and trailers. The analyzed sequences carried a CRISPR1-Cas type II-A system with four *cas* genes (*cas9*, *cas1*, *cas2*, and *csn2*). The number of spacers ranged from three to 32 per isolate, corresponding to a CRISPR1 array size of 434 to 2348 bp. In addition to CRISPR1, seven isolates (ST12, n = 5; ST10, n = 1; ST24, n = 1) concurrently harbored a CRISPR2 system, confirming its rare presence. The number of CRISPR2 spacers ranged from 4 to 10 per isolate, with array sizes ranging from 497 to 896 bp. All sequenced CRISPR2-Cas type I-C systems contained seven *cas* genes (*cas3, cas5c, cas8c, cas7c, cas4, cas1*, and *cas2*).

In line with these results, 2,835 publicly available genomes from other African countries (Angola, Botswana, Ethiopia, Gambia, Kenya, Malawi, Mozambique, Nigeria, South Africa) were analyzed for the presence of the *csn2* gene, which is specific to type II-A systems and indicative of CRISPR1 (Makarova et al., 2020). The *csn2* gene is very conserved and was identified by *in silico* PCR in over 99% (2,825 of 2,835) of the analyzed African isolates.

A total of 1,441 spacers were identified, of which 287 spacers were unique (275 in CRISPR1 and 12 in CRISPR2) appearing only once among the analyzed genomes. Unique spacer sizes ranged from 27 to 36 bp (**Supplementary Table S4**). The remaining 1,154 spacers were shared and duplicated within or across isolate (1125 in CRISPR1 and 29 in CRISPR2), corresponding to 258 distinct spacers after removing duplicates (**Supplementary Table S5**) ranging from 28 to 35 bp. Frequently repeated spacer sequences are listed in **Table 2**. Internal spacer duplication(s) within the same isolate was observed only in the CRISPR1 arrays but not in CRISPR2. Spacer duplication(s) in the CRISPR1 arrays within the same genome were observed in 37 isolates, including ST1 (n = 13), ST19 (n = 8), ST12 (n = 4), ST23 (n = 3), and ST932 (n = 2), and single isolates of STs 3, 6, 14, 25, 196, 556, and 934.

**Table 2:** The topmost repeated spacers within the same isolate and/or among more than one isolate of colonizing GBS strains in Egypt.

Spacer	Frequency
TACTTGACGAATTGAAGATGACGGAATTTA	21
ACTCTAAATGATAGTTATGAGTTAAATGTT	18
TAAATTCCGTCATCTTCAATTCGTCAAGTA	17
AACATTAGCCTTTTCTAACTCTTCAGCTGT	15
TTAACAGTTTCAAGTCTGTCTTGTTACTTA	15
CCGTCAAACAAGAGCGACAGCGAAACAAGC	13
ATATGTTCCACTCTATGAATTTAGGCTCAT	12
TATGTCTTCTAACAGTTGCTTCTTGTGCTT	12
AACATTTAACTCATAACTATCATTTAGAGT	11
CAAATTACAGTTTCGACTGATTATGGAAAT	11
GATTACCTTAGATGATGTTCTAATCGGTAA	11
TCTTCTTTTTAATTCTTCTAACACTCCATC	11
AAAACGTCGTAAACGTGTCATTGATTGTAT	10
TTTAAAGAGATATCTGTTTCATCTTGCGGA	10

**Figure 1:**
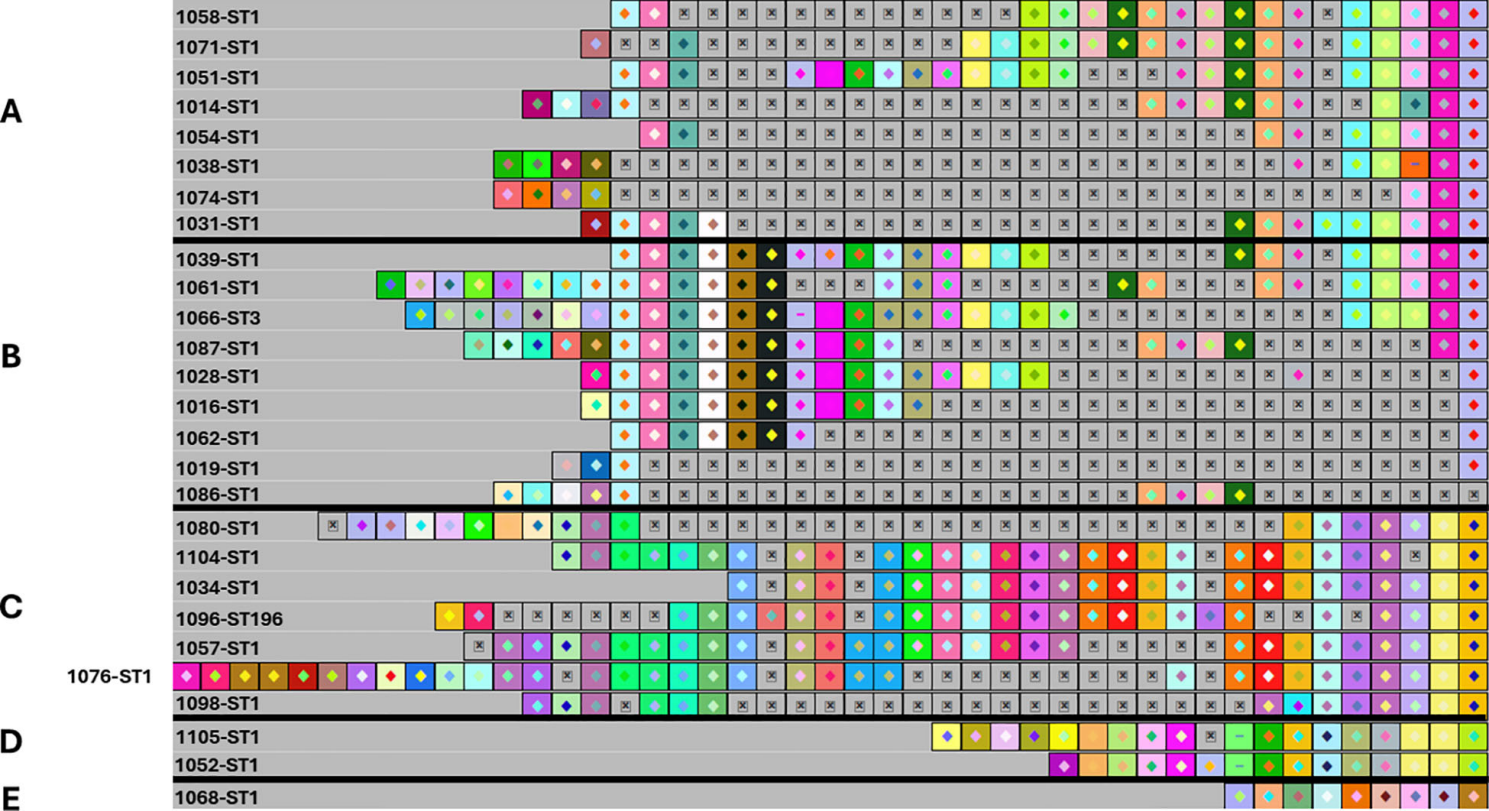
The CRISPR1 arrays of ST1, ST3, and ST196 isolates showing CC1 subgroups from 1 to 5. **(A)** CRISPR1 array of CC1 subgroup 1. **(B)** CC1 subgroup 2. **(C)** CC1 subgroup 3. **(D)** CC1 subgroup 4. **(E)** CC1 subgroup 5. According to eBURST, ST3 and ST196 belonged to different CCs. However, these were grouped into CC1 based on their homologous CRISPR1 array to ST1. The CRISPR1 arrays are represented using CRISPRViZ. A black line separates CRISPR1 subgroups. Spacers are represented as colored boxes. Gaps (=missing spacers) are shown with a boxed cross symbol (⊠) after alignment of identical spacers between strains of the same group. Arrays are oriented with respect to the leader sequence located on the left.

**Figure 2:**
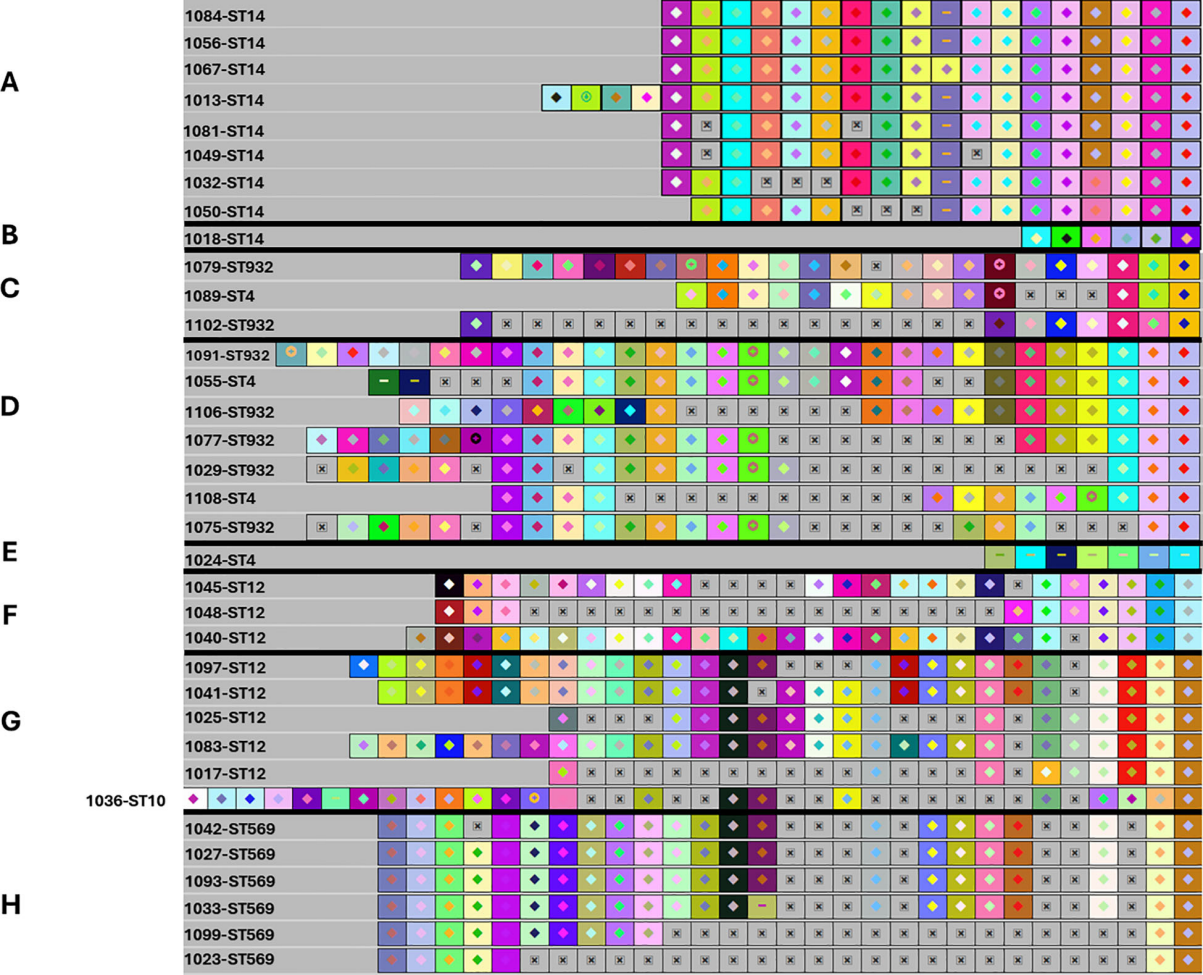
The CRISPR1 arrays of ST14/CC1, CC4 (ST4 and ST932), CC12 (ST12 and ST10) isolates, and the singleton ST569. A-B. The CRISPR1 arrays of ST14/CC1 isolates showing CC1 subgroups 6 and 7. **(A)** CRISPR1 array of CC1 subgroup 6. **(B)** CRISPR1 array of CC1 subgroup 7. All except one of the ST14 isolates were gathered into a single cluster. C-E. The CRISPR1 arrays of CC4 (ST4 and ST932) isolates showing CC4 subgroups from 1 to 3. **(C)** CRISPR1 array of CC4 subgroup 1. **(D)** CRISPR1 array of CC4 subgroup 2. **(E)** CRISPR1 array of CC4 subgroup 3. STs 4 and 932 were grouped into two clusters, with one ST4 isolate having a distinct array. **(F-H)** The CRISPR1 arrays of CC12 (ST12 and ST10) isolates and the singleton ST569. **(F)** CRISPR1 array of CC12 subgroup-1. **(G)** CRISPR1 array of CC12 subgroup-2. **(H)** CRISPR1 array of ST569 isolates. The CRISPR1 arrays are represented using CRISPRViZ. A black line separates CRISPR1 subgroups. Spacers are represented as colored boxes. Gaps (=missing spacers) are shown with a boxed cross symbol (⊠) after alignment of identical spacers between strains of the same group. Arrays are oriented with respect to the leader sequence located on the left.

**Figure 3:**
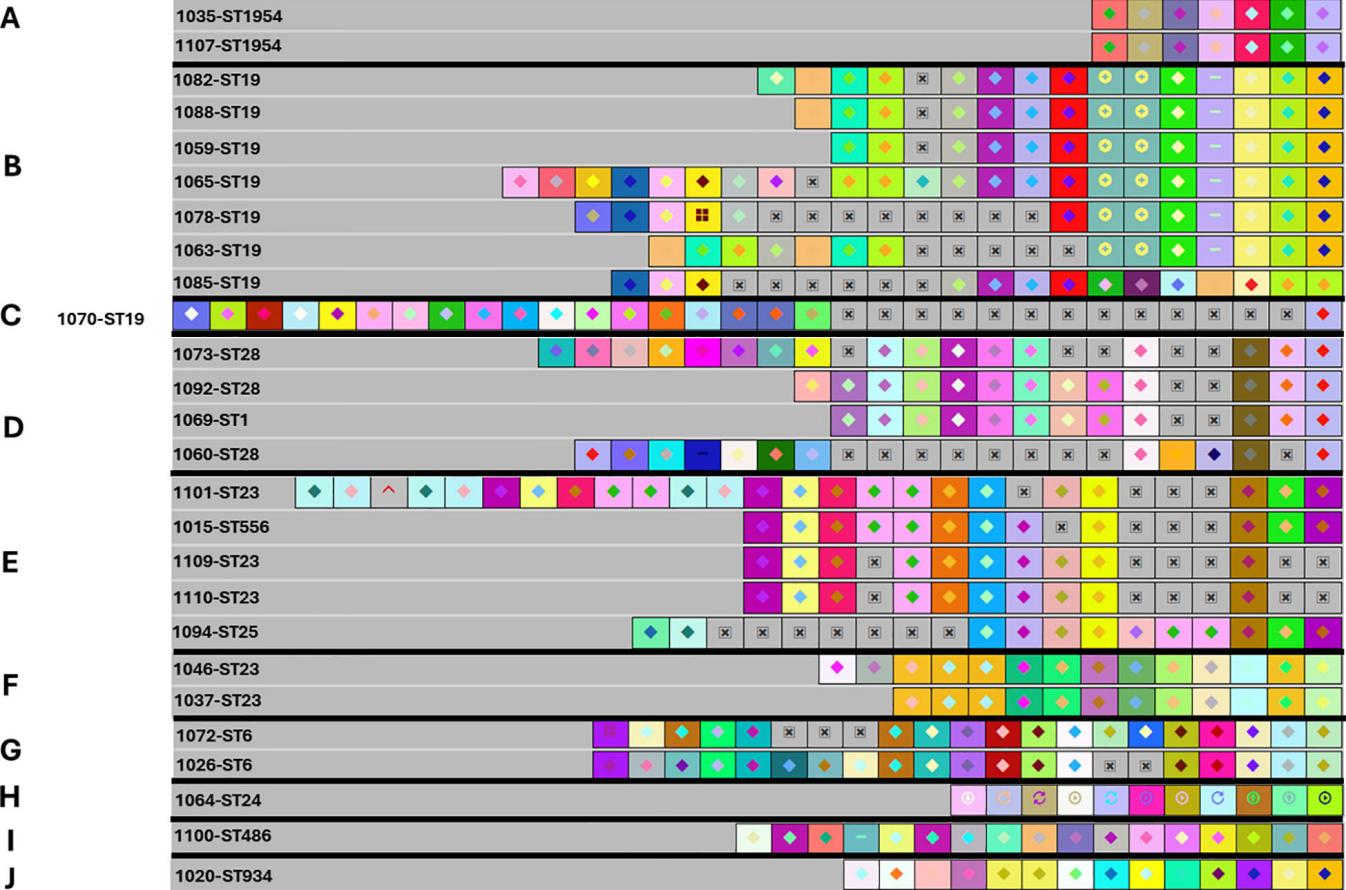
The CRISPR1 arrays of the novel ST1954/CC17, STs 19, 28, and 1 isolates, CC23 (ST23, ST25, ST556), and the singletons ST6, ST24, ST486, and ST934 isolates. **(A)** The CRISPR1 arrays of the novel ST1954/CC17 isolates. B-D. The CRISPR1 arrays of STs 19, 28, and 1 isolates showing CC19 subgroups from 1 to 3. **(B)** CRISPR1 array of CC19 subgroup 1. **(C)** CRISPR1 array of CC19 subgroup 2. **(D)** CRISPR1 array of CC19 subgroup 3. All except one of the ST19 isolates were gathered into a single cluster. The ST28 isolates were grouped with a single ST1 isolate showing a CRISPR1 array homologous to the ST28 cluster. E-F. The CRISPR1 arrays of CC23 (ST23, ST25, ST556) isolates showing CC23 subgroups 1 and 2. **(E)** CRISPR1 array of CC23 subgroup 1. **(F)** CRISPR1 array of CC23 subgroup 2. The CC23 isolates were grouped into two clusters. G-J. The CRISPR1 arrays of the singletons ST6, ST24, ST486, and ST934 isolates. **(G)** CRISPR1 array of ST6 (n = 2). **(H)** CRISPR1 array of ST24 (n = 1). **(I)** CRISPR1 array of ST486 (n = 1). **(J)** CRISPR1 array of ST934 (n = 1). The CRISPR1 arrays are represented using CRISPRViZ. A black line separates CRISPR1 subgroups. Spacers are represented as colored boxes. Gaps (=missing spacers) are shown with a boxed cross symbol (⊠) after alignment of identical spacers between strains of the same group. Arrays are oriented with respect to the leader sequence located on the left.

**Figure 4:**
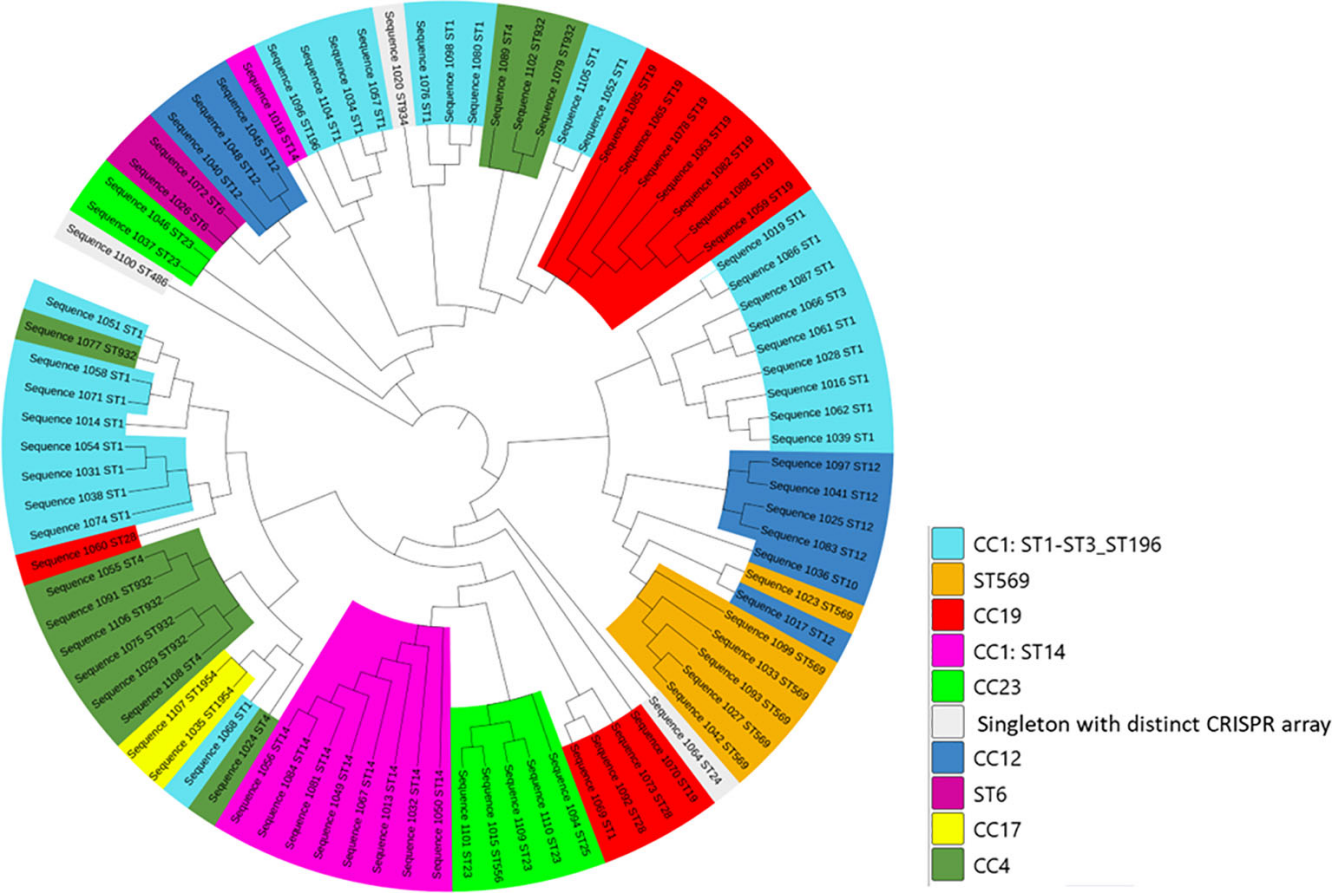
Phylogenetic circular tree of the CRISPR1 sequences of colonizing GBS strains from Egypt. The phylogenetic tree of CRISPR1 loci was built using the Interactive Tree Of Life (iTOL).

Using CRISPRViZ isolates were aligned and grouped based on CRISPR1 array homology (**Figures 1**–**3**). Strong congruence was observed between CRISPR1 array clustering and MLST typing. A total of 81 isolates were grouped into 16 CRISPR1 clusters, whereas seven isolates demonstrated distinct arrays and were not grouped into any cluster. These were then regrouped according to MLST type. For CC1 (**Figures 1A–E**), STs 1, 3, and 196 were grouped into four clusters, with one isolate showing a distinct array (CC1 subgroup-1 to 5). Although eBURST assigned ST3 and ST196 to different CCs, their CRISPR1 homology grouped them under ST1. All except one of the ST14 isolates were clustered together (CC1 subgroup-6 to 7) (**Figures 2A, B**). For CC4 (**Figures 2C–E**), rare ST4s and African-specific ST932 (Shabayek et al., 2022) formed two clusters, whereas one ST4 isolate had a distinct array (CC4 subgroups 1 to 3). CC12 (**Figures 2F, G**) comprised ST12 and ST10 isolates in two clusters (CC12 subgroups 1 to 2). For CC17 (**Figure 3A**), the CRISPR1 arrays of the two novel ST1954/HvgA positive/serotype III isolates (Shabayek et al., 2022), were identical, demonstrating a perfect match with 100% similarity, and were gathered into a single cluster. For CC19 (**Figures 3B–D**), all except one of the ST19 isolates were clustered together and ST28 isolates were grouped with a single ST1 isolate showing a CRISPR1 array homologous to the ST28 cluster (CC19 subgroups 1 to 3). CC23 (**Figures 3E, F**) grouped the STs 23, 25, and 556 in two clusters (CC23 subgroup-1 to 2). ST6 and the African-specific ST569 isolates (Shabayek et al., 2022) formed separate singleton clusters (**Figures 2H**, **3G**). ST24 and African-specific STs 486 and 934 (Shabayek et al., 2022) had distinct CRISPR1 arrays and were not grouped into any cluster (**Figures 3H–J**). Notably, isolates with unique CRISPR1 arrays had 66% to 100% unique spacers as shown by CRISPRCasFinder. A phylogenetic tree of the analyzed CRISPR1 loci is shown in **Figure 4**.

A comparison of the spacers comprising the CRISPR1 loci revealed extreme diversity (**Figures 1**–**3**). A total of 87 different spacer profiles were identified, confirming that the GBS CRISPR1 locus is highly dynamic. Spacers near the trailer end were more conserved, whereas those near the leader end were mostly unique or rare. However, some isolates showed spacer deletions at the trailer end and some isolates lacked unique spacers in the leader region. These spacers were in the middle part of the CRISPR array of related strains. Events involving several spacer deletions in the middle part of the CRISPR1 array without the acquisition of unique spacers or duplication of conserved spacers were also observed, as well as internal spacer switches.

Finally, using CRISPRCasFinder, we analyzed the flanking regions of CRISPR1 and CRISPR2 (leader and trailer ends), as well as the DR, as these sequences sometimes show variation between groups of isolates. A total of four DR sequences were identified in the CRISPR1 loci (**Supplementary Table S6**); the two most prevalent, 36 bp each, appeared in 64% (n = 58) and 29% (n = 26) of isolates. The other two, 37 bp long, were found in ST19 (n=2) and the novel ST1954/CC17 (Shabayek et al., 2022) (n = 2). For the seven CRISPR2 loci, two 32-bp DR sequences were identified. Concerning leader sequences, a total of nine sequences directly adjacent to the first CRISPR1 repeat were identified (**Supplementary Table S7**). The three most common CRISPR1 leader sequences were found in 57% (n = 51), 20% (n = 18), and 9% (n = 8) of the isolates, respectively. For the CRISPR2 loci, two leader sequences were identified. Concerning trailer sequences, nine sequences located downstream of CRISPR1 were identified (**Supplementary Table S8**). The three most common trailer sequences were found in 41% (n = 37), 31% (n = 28), and 13% (n = 12) of the isolates, respectively. For the CRISPR2 loci, two trailer sequences were identified.

**Figure 5:**
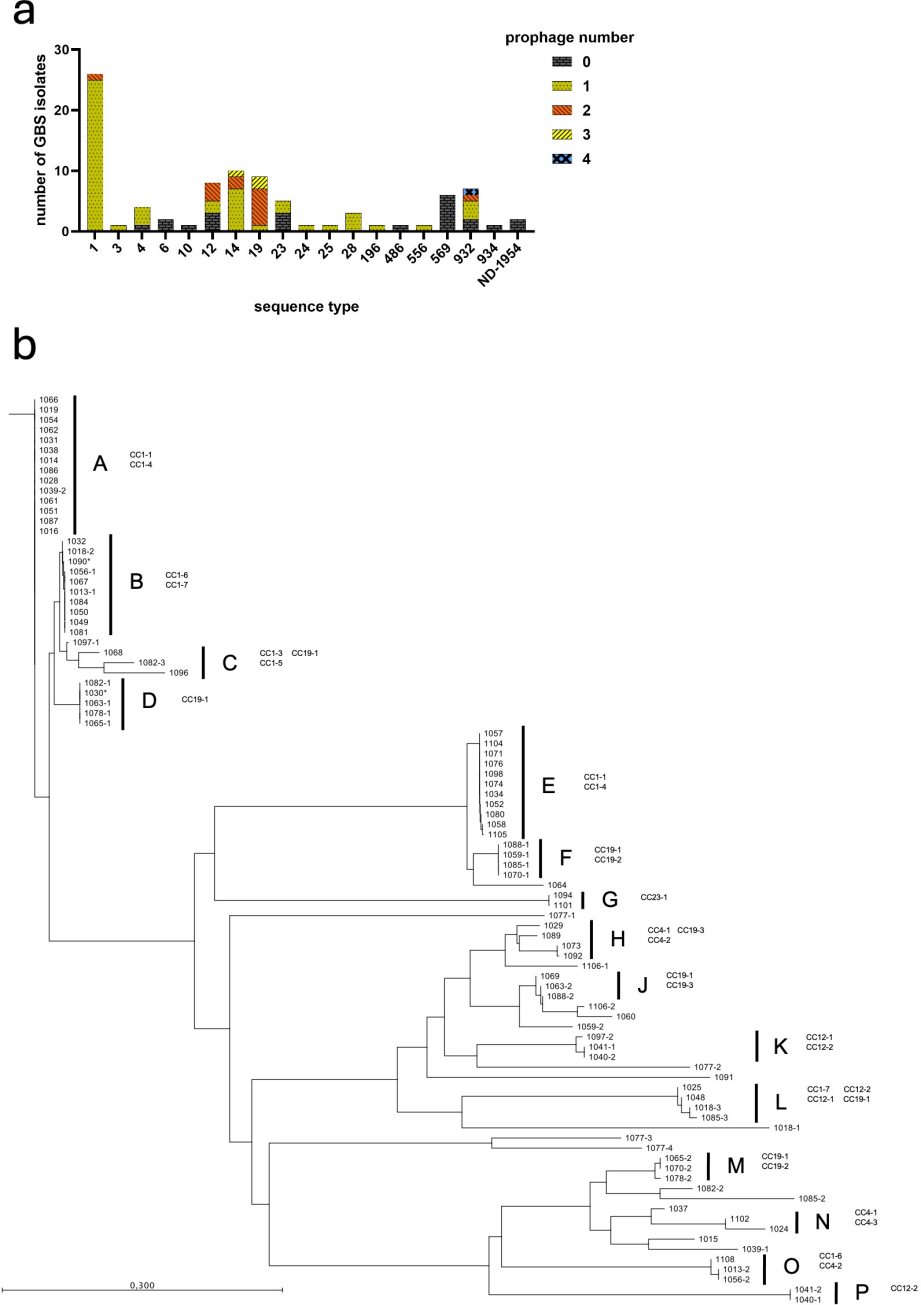
Distribution and phylogeny of GBS prophages. **(a)** Prophage distribution across sequence types of Egyptian GBS isolates. **(b)** Neighbor Joining tree constructed on the genome comparison at nucleotide level of prophages found in Egyptian GBS strains by bootstrap analysis using Qiagen CLC Main Workbench 7.7.3. Multiple-sequence alignment was constructed using Qiagen CLC Main Workbench 7.7.3 with default settings. Assigned prophage groups from (A-P) are depicted as well as corresponding CRISPR1 array clusters. Prophages labeled with an asterisk originate from GBS strains not included in CRISPR1 array clustering.

CRISPR-Cas systems provide protection against phage invasion, to investigate how CRISPR profiles influence prophage content in the Egyptian GBS population, the assembled whole-genome sequences were analyzed with PHASTEST (Penadés et al., 2015). In total, 149 prophage sequences were identified across 90 GBS isolates. A total of 61 prophages were excluded from analysis since they were predicted as “incomplete” or “questionable” by PHASTEST. The number and size of intact prophages per genome are shown in **Table 3**. Among the analyzed GBS genomes, most (56.7%, 51/90) carried only one prophage, 14.4% (13/90) had two, 3.3% (3/90) had three, and 1.1% (1/90) four. No prophages were detected in 24.4% of the GBS isolates. The prevalence of prophages varied across different STs (**Figure 5a**). Isolates of ST1, ST4, and ST28 mainly carried one prophage, whereas ST12, ST14, ST19, and the African-specific ST932 showed more heterogeneity. Interestingly, no prophages were detected in the African-specific ST569.

**Table 3:** Prophages in Egyptian GBS isolates.

Isolate	ST	# of prophage	Prophage size (kbp)	Prophage cluster	CRISPR-Cas system	# of spacers	CRISPR1 clustering
1058	1	1	52	E	II-A	16	CC1-1
1071		1	52.4			18	
1074		1	51.9			6	
1014		1	52	A		13	
1054		1	52.7			8	
1038		1	52.2			9	
1051		1	52			22	
1031		1	52.2			13	
1039	1	2	45.8	None	II-A	22	CC1-2
			52	A			
1061		1	52			25	
1086		1	52			8	
1087		1	51.8			20	
1028		1	52			17	
1016		1	51.8			12	
1062		1	52.2			7	
1019		1	52			3	
1066	3	1	52			27	
1080	1	1	52.4	E	II-A	16	CC1-3
1104		1	52.4			27	
1034		1	52.4			22	
1098		1	51.9			13	
1057		1	52.2			27	
1076		1	51.9			32	
1096	196	1	55.6	C		25	
1105	1	1	38.7	E	II-A	18	CC1-4
1052		1	52.4			15	
1068	1	1	43.1	C	II-A	8	CC1-5
1084	14	1	43.1	B	II-A	17	CC1-6
1056		2	43.1	B		17	
			52.2	O			
1067		1	43.1	B		17	
1013		2	43.1	B		21	
			52.2	O			
1081		1	43.1	B		15	
1049		1	43.1	B		15	
1032		1	52	B		14	
1050		1	43.1	B		13	
1018	14	3	36.6	None	II-A	5	CC1-7
			52	B			
			38.4	L			
1079	932	0	–	–	II-A	22	CC4-1
1089	4	1	58.1	H		13	
1102	932	1	43.8	N		7	
1091	932	1	32.2	None	II-A	29	CC4-2
1055	4	0	–	–		21	
1106	932	2	55	None		19	
			24.9	None			
1077	932	4	34.7	None		22	
			44.8	None			
			25	None			
			25.9	None			
1029	932	1	59.5	H		15	
1108	4	1	39.7	O		12	
1075	932	0	–	–		18	
1024	4	1	72.7	N	II-A	6	CC4-3
1045	12	0	–	–	II-A, I-C	21, (6)	CC12-1
1048		1	38.7	L		9, (4)	
1040		2	57.9	P	II-A	26	
			47.4	K			
1097	12	2	57.9	None	II-A	25	CC12-2
			47	K			
1041	12	2	57.9	K		26	
			47.4	P			
1025	12	1	38.7	L	II-A, I-C	15, (4)	
1083	12	0	–	–		28, (6)	
1017	12	0	–	–		8, (4)	
1036	10	0	–	–		22, (7)	
1042	569	0	–	–	II-A	20	Singleton 569
1027		0	–	–		21	
1033		0	–	–		21	
1093		0	–	–		21	
1099		0	–	–		11	
1023		0	–	–		6	
1035	1954	0	–	–	II-A	6	CC17
1107		0	–	–		6	
1082	19	3	50.9	D	II-A	14	CC19-1
			44.8	None			
			44	C			
1088		2	50.9	F		13	
			48.8	J			
1059		2	50.9	F		12	
			49.4	None			
1065		2	50.9	D		21	
			49.5	M			
1078		2	50.9	D		12	
			53.1	M			
1063		2	50.9	D		13	
			48.8	J			
1085		3	50.9	F		14	
			53.3	None			
			50.2	L			
1070	19	2	50.9	F	II-A	18	CC19-2
			49.5	M			
1073	28	1	57.8	H	II-A	16	CC19-3
1092	28	1	58.5	H		12	
1069	1	1	62.4	J		11	
1060	28	1	16.4	None		11	
1101	23	1	46.1	G	II-A	23	CC23-1
1015	556	1	47.3	None		12	
1109	23	0	–	–		10	
1110	23	0	–	–		10	
1094	25	1	46.2	G		12	
1046	23	0	–	–	II-A	13	CC23-2
1037		1	45.7	None		11	
1072	6	0	–	–	II-A	17	Singleton 6
1026		0	–	–		18	
1064	24	1	39.7	None	II-A, I-C	17, (10)	Singleton 24
1100	486	0	–	–	II-A	17	Singleton 486
1020	934	0	–	–	II-A	13	Singleton 934

Based on the alignment and the following constructed phylogenetic tree (**Figure 5b**), the identified prophages were assigned into prophage clusters A-P (**Table 3**). Clusters of prophage sequences with ≥75% identity were aligned (**Table 1**; **Supplementary Table S3**) and further compared with the NCBI nr/nt database of viruses and bacteria using MEGABLAST. Most resembled annotated Streptococcus phages, including phages of *S. agalactiae*, *S. dysgalactiae*, and *S. pyogenes*, and Enterococcus phages (**Tables 1**, **4**). The 14 analyzed prophages from cluster A matched *S. agalactiae* phage Javan55 with 100% identity over 66%-67% genome coverage (Juhas, 2015). The 11 prophages from cluster E also matched Javan55 with 100% identity over a coverage of 67%, but had ≤50% mutual similarity to cluster A prophages. Prophages from cluster D and F matched *S. agalactiae* phage Javan29, each showing 100% identity and 68% genome coverage (Juhas, 2015); however, the mutual similarity of both clusters was ≤50%. Cluster B, J, and L prophages resembled *S. agalactiae* phage Javan51, Javan39, and Javan23, respectively (Juhas, 2015). Prophages of clusters G and P did not match to any annotated bacteriophage. Despite their mutual similarity of at least 87%, cluster H prophages did not consistently match a particular phage (**Table 4**). Cluster C, K, M, N, and O prophages matched various known phages including *S. agalactiae* phages phiStag1_20280_6_181 (Russell et al., 1969), vB_Sags-UPM1 (van der Mee-Marquet et al., 2018), phiGBSVK-D_GBSInt2.2 (Salloum et al., 2011), LF2 (Domelier et al., 2009), and phiGBSVK-F2_GBSInt11.1 (Salloum et al., 2011). **Table 4** lists unclustered prophages and their closest annotated matches.

**Table 4:** Similarity to previously characterized phages of prophage cluster H and prophages not clustered.

Prophage	Phage hit	Coverage	Identity
Prophage cluster H			
1029	phiGBSVK-E2_GBSInt11.1	41%	97%
1073	Javan37	46%	99.4%
1089	Javan7	45%	95.45%
1092	Javan37	46%	99.31%
Prophages without cluster			
1015	vB_Sags-UPM1	52%	86.8%
1018-1	Javan11	75%	98.17%
1037	LF2	63%	89.3%
1039-1	phiGBSVK-E1_GBS11.1	46%	95.97%
1060	phiGBSVK-D_GBSInt1	100%	99.99%
1059-2	phiGBSVK-D_GBSInt6.2	54%	91.39%
1064	phiStag1_20280_6_181	96%	100%
1077-2	Javan149^-^	88%	99.96%
1077-3	vB_EfaS_IME197^#^	77%	97.26%
1077-4	VEsP-1^#^	65%	93.54%
1082-2	phiGBSVK-D_GBSInt2.1	79%	91.96%
1085-2	Javan52	36%	98.4%
1091	Javan478^+^	23%	84.75%
1097-1	Javan8	95%	99.86%
1106-1	phiGBSVK-E2_GBSInt11.1	37%	97%
1106-2	LF2	93%	89.17%

Unless otherwise stated these are GBS phages. ^+^ indicates *S. pyogenes* phage; ^-^ indicates *S. dysgalactiae* phage; ^#^ indicates *Enterococcus faecalis* and *Enterococcus* sp. phage.

Prophage pattern correlated with the CRISPR1 clustering (**Table 3**; **Figure 5b**). Prophage clusters A and E were exclusive to GBS isolates of CRISPR1 cluster CC1-1–CC1-4; cluster B and O prophages to CC1–6 and CC1-7; prophage clusters D, F, J, and M to CC19-1, CC19-2, and CC19-3; cluster G prophages to CC23-1, K to CC12–1 and CC12-2; and prophages N and P to CC4–1 and CC4–2 or CC12-2, respectively; clusters C, H, and L spanned multiple CRISPR1 groups. No prophages were detected In CRISPR1 clusters CC17, singleton ST569, and singleton ST6.

## Discussion

CRISPR plays a key role in adaptive immunity against foreign DNA sequences such as integrative and conjugative elements, phages, and plasmids (Lopez-Sanchez et al., 2012). We investigated the structure and content of CRISPR1 and CRISPR2 loci in a collection of 90 whole-genome sequenced colonizing GBS isolates from healthy Egyptian women. Our analysis confirmed the ubiquitous nature of CRISPR1 and revealed its high diversity and dynamism, whereas CRISPR2 was rare, less diverse, and less active, as previously reported (Lopez-Sanchez et al., 2012; Lier et al., 2015; Beauruelle et al., 2017; Beauruelle et al., 2021). In line with earlier findings (Lopez-Sanchez et al., 2012; Lier et al., 2015; Beauruelle et al., 2017; Beauruelle et al., 2021), we observed a strong congruence between CRISPR1-based genotyping and MLST, underscoring the utility of CRISPR typing in characterizing the population structure of vaginally carried GBS. The CRISPR1 spacer content efficiently reflected the GBS population structure. To assess the applicability of CRISPR typing in African GBS isolates more specifically, *in silico* PCR was performed, screening for the presence of the *csn2* gene, which is exclusive to CRISPR-Cas type II-A systems (Makarova et al., 2020). Among 2835 *S. agalactiae* genomes, collected from nine different African countries, the type II-A system was easily detected in over 99% of genomes, demonstrating the widespread presence of this system and highlighting CRISPR typing as a robust tool for GBS population surveillance.

Lopez-Sanchez et al., 2012 (Lopez-Sanchez et al., 2012) demonstrated that CRISPR analysis not only enables GBS typing based on ancient spacers but also facilitates subtyping through the analysis of more recently acquired spacers, which is an even more effective method, as it allows clustering of isolates based on newer spacers. This precise subtyping may have significant epidemiological implications. For instance, we previously reported that serotype VI, a common lineage among our isolates, was almost exclusively associated with ST-14 (Shabayek et al., 2022). In the current study, we showed that all ST14 isolates, except one, clustered together based on CRISPR1 profiles. This isolate was the only ST14 isolate carrying serotype III and harboring both Alp2 and Epsilon surface proteins, whereas all other ST14 isolates carried serotype VI and harbored only the Epsilon surface protein. Furthermore, CRISPR1 clustering grouped ST28 isolates with a single ST1 isolate, all of these belonged to serotype II and harbored the Rib surface protein. Interestingly, all African-specific STs (486, 569, 932, 934) were clearly distinguishable from other lineages based on CRISPR1 profiles. Lopez-Sanchez et al. (2012) (Lopez-Sanchez et al., 2012) also suggested that CRISPR1 analysis could support subtyping according to geographical distribution. Therefore, CRISPR1-based subtyping may be useful for tracking regions specific GBS lineages and monitoring their global circulation.

CRISPR diversity is driven by spacer acquisition, deletion, and duplication, reflecting the activity of this adaptive immune system. In concordance with previous studies (Lopez-Sanchez et al., 2012; Lier et al., 2015; Beauruelle et al., 2017; Beauruelle et al., 2021), we observed that spacer acquisitions frequently occurred near the leader end of the CRISPR array. Consistent with Beauruelle et al. (2017) (Beauruelle et al., 2017), isolates showing polarized acquisitions patterns predominantly belonged to ST1. Additionally, previous studies (Deveau et al., 2008; Horvath et al., 2008; Lopez-Sanchez et al., 2012; Beauruelle et al., 2017) have shown that spacer deletions typically occur in the internal region of the array and are rarely found near the trailer end. Our findings support this, as most spacer deletions were observed in the internal region of the CRISPR locus. However, we also identified spacer deletions near the trailer end in a few ST23 isolates. Furthermore, we found that internal spacer duplication(s) were more frequent among the CC1 isolates—a pattern reported among CC23 isolates by Beauruelle et al (Beauruelle et al., 2017). Interestingly, identical spacer loci were observed only in CC17 isolates in our study, consistent with earlier findings (Lopez-Sanchez et al., 2012). This aligns with a study from 2017 (Beauruelle et al., 2017) that found no modifications in CRISPR1 loci among CC17 isolates. Similarly, Beauruelle et al. (2021) (Beauruelle et al., 2021) reported fewer spacer variations at the CRISPR1 locus among CC17 isolates, attributing this to the presence of highly homogenous and prevalent CC17 clones.

Spacer polymorphisms revealed varying degrees of heterogeneity among the ST and CC groups. In concordance with Lier et al. (2015) (Lier et al., 2015) and Beauruelle et al. (2021) (Beauruelle et al., 2021), we observed CRISPR1 clusters containing ST1, and to a lesser extent, those containing CC4, CC12, and CC19 were more heterogeneous, as evidenced by their separation into several subgroups. According to Da Cunha and colleagues (Da Cunha et al., 2014), this heterogeneity might be explained by a higher rate of recombination within certain CCs. In contrast, we found that CRISPR1 clusters containing the novel ST1954/CC17 clones and CC23 isolates were more homogenous. This finding is consistent with previous studies (Lier et al., 2015; Beauruelle et al., 2017; Beauruelle et al., 2021) which reported the lowest CRISPR1 polymorphism in CC17 followed by CC23. Moreover, Beauruelle et al. (2017) reported that CC23 isolates shared a relatively high number of common spacers. We observed a similar pattern of homogeneity in CRISPR1 clusters containing ST14 and the African-specific ST569. Beauruelle et al. (2021) and Lier et al. (2015) (Lier et al., 2015; Beauruelle et al., 2021) proposed that the level of spacer diversity in a CRISPR array is a useful indicator of the functional activity of the associated locus. CRISPR polymorphism also reflects a strain’s ability to acquire new spacers. Lier et al. (2015) (Lier et al., 2015) identified CC17 isolates as having significantly fewer spacers compared with other CCs.

Prophages contribute to virulence, pathogenicity, and the genomic diversity of GBS (Penadés et al., 2015; Davies et al., 2016).

They are typically acquired through horizontal gene transfer and play a major role in the acquisition of bacterial virulence factors. The integration and excision of prophages strongly influence evolution of pathogenic bacteria and may facilitate adaptation to new hosts and environmental challenges. However, the initiation of a phage lytic cycle through environmental stress kills the bacterial host, and the bacterial immunity system CRISPR-Cas provides sequence-specific immunity against phages. The presence of certain phages and the pattern of a specific phage content may thus be associated with distinct CRISPR profiles. Therefore, in addition to CRISPR profiling, the 90 Egyptian GBS isolates were analyzed to determine the presence and diversity of prophages. In total, 149 prophages were identified with most isolates harboring at least one integrated prophage and some genomes carrying multiple prophages. Among the 90 analyzed GBS isolates, 68 (∼76%) had at least one prophage integrated, with 13 isolates carrying two prophages, 3 isolates carrying three prophages, and 1 isolate carrying four prophages. Consistent with other studies on GBS prophages, we observed differences in prophage content between isolates of different STs (**Figure 5a**) (Salloum et al., 2011; van der Mee-Marquet et al., 2018). When analyzing the prophage content in relation to different CRISPR types, we also observed associations between certain prophages and specific CRISPR types, supporting the potential of CRISPR typing to efficiently differentiate distinct lineages within the GBS population. Among the prophage carriers, the highest prophage content was observed in ST19 (2.1 average prophages per genome) whereas the lowest was found in ST23 (0.4 prophages per genome). This variation may be associated with CRISPR locus activity, as an inverse correlation between prophage content and CRISPR spacer number has been reported in *S. pyogenes* (Nozawa et al., 2011). The integration of multiple prophages from different clusters into the bacterial genome may enhance genomic diversity, virulence, or fitness and appears to be linked to specific CRISPR profiles potentially conferring selective advantages to the host bacterium (Ramisetty and Sudhakari, 2019).

Vaccine development requires careful consideration of geographic variations in the population structure of GBS. The World Health Organization (WHO) has declared GBS vaccination among pregnant women as a priority in low- and middle-income countries (Seale et al., 2019). CRISPR typing offers a low-cost and simple alternative to MLST, as it requires analysis of only one locus, whereas MLST involves sequencing seven loci (Radtke et al., 2010; Sabat et al., 2013; Beauruelle et al., 2021). CRISPR typing has proven effective in evaluating the diversity and evolutionary dynamics of GBS vaginal carriage (Beauruelle et al., 2021). As previously suggested (Beauruelle et al., 2021), this method seems particularly valuable in middle- and low-income countries where data on the molecular characteristics of GBS isolates remain limited.

## Conclusions

CRISPR1-based typing provides molecular-level insights into the population structure of GBS isolates rom vaginal carriage. In this study, we observed a strong congruence between MLST, CRISPR typing, and phage content in Egyptian GBS isolates. Compared with MLST, CRISPR typing appears to be a low-cost alternative with great discriminatory power, making it a valuable tool investigating GBS population dynamics. CRISPR1 diversity is driven by spacer acquisition, deletion, and duplication, reflecting the activity and adaptability of this protection system. Isolates with polarized acquisitions particularly those belonging to ST1 exhibited more heterogenous spacer profiles. In contrast, the novel ST1954/CC17 isolates showed a lower ability to acquire novel spacers and were the only clones with identical spacer profiles. These findings suggest that the evolution of CRISPR1 arrays may be linked to the dynamics of phylogenetic lineages. Given the widespread presence of CRISPR-Cas type II-A systems, CRISPR typing should be applicable to the vast majority of African GBS isolates. Notably, all African-specific STs in our study could be discriminated from other lineages. Subtyping based on the restricted phylogeographic distribution of specific lineages appears feasible. In summary, CRISPR1 typing is a simple and effective method to monitor the circulation of GBS clones at both regional and global scales.

## Data Availability

The datasets presented in this study can be found in online repositories. The names of the repository/repositories and accession number(s) can be found below: https://www.ebi.ac.uk/ena, **Supplementary Table S2**.
